# Measuring frailty and its association with key outcomes in the ambulance setting: a cross sectional observational study

**DOI:** 10.1186/s12877-022-03633-z

**Published:** 2022-12-05

**Authors:** Karl Charlton, David R Sinclair, Barbara Hanratty, Emma Burrow, Daniel Stow

**Affiliations:** 1 North East Ambulance Service NHS Foundation Trust, Ambulance HQ, Bernicia House, Goldcrest Way, Newburn Riverside, Newcastle upon Tyne, NE15 8NY UK; 2 Population and Health Sciences Institute, Newcastle University, Newcastle upon Tyne, NE2 4AX UK; 3 Wolfson Institute of Population Health, Barts & The London School of Medicine & Dentistry, Queen Mary University of London, London, E1 4NS UK

**Keywords:** Frailty, Urgent care, Ambulance, Epidemiology, Public health

## Abstract

**Background:**

Little is known about frailty in the ambulance setting, or its association with outcomes relevant to ambulance services. We sought to measure frailty in people aged ≥ 50 attended by an ambulance, and describe the relationship between frailty, odds of conveyance to hospital, and duration at scene.

**Methods:**

An observational study between 01/01/2021-30/06/2021 in North East Ambulance Service, England. Participants were aged ≥ 50 attended by an ambulance, excluding patients requiring immediate treatment for a life-threatening condition or with Glasgow Coma Scale < 15. Paramedics (*n* = 112) measured patient frailty using the Clinical Frailty Scale (CFS). Additional information was extracted from ambulance care records. Weighted regression models examined associations between frailty, hospital conveyance, and duration at scene.

**Results:**

Three thousand and fifty-six callouts were observed (mean patient age: 78.1 years, 57.2% female). Frailty prevalence (CFS ≥ 5) was 58.7%. Median duration at scene was 47.0 min (interquartile range 34.0–67.0 min). Ambulances spent a median of 8.2 (95%CI:5.4–11.0) minutes longer with frail patients than non-frail patients. Frail patients were less likely to be conveyed to hospital than non-frail patients (OR:0.75, 95%CI:0.60–0.94).

**Conclusion:**

Frailty is common among people aged ≥ 50 attended by an ambulance and an important influence on workload. Ambulance services need a good understanding of frailty to meet patient needs. As populations age, community support should be prioritised to deliver appropriate frailty care and reduce demands on ambulance services.

## Background

National Health Service (NHS) ambulance services are at the heart of the urgent and emergency care system in the United Kingdom (UK) [1]. In addition to mobile emergency and primary care, they provide a vital interface to other health and social services [2]. Older populations access healthcare through ambulance services at disproportionally higher rates than younger populations [3], and demand for ambulance services is projected to increase as populations age [4].

Frailty is a distinct health state associated with the ageing process. It has been described as one of the most problematic expressions of population ageing [5], presenting increasing challenge to health systems [6, 7]. The association between frailty, poorer clinical outcomes and higher healthcare resource use is well established across multiple healthcare settings [8, 9].

In urgent care settings, frailty assessment is advocated for use to enhance patient-centred care and clinical outcomes [10]. However, there is little evidence on frailty in the ambulance setting, where paramedics make on-the spot decisions about who to treat at the scene or transfer to other care settings (including urgent care and the emergency department (ED)). The extent of frailty in the population of ambulance service patients is unclear, and the specific needs of older populations using the ambulance service are not well understood [11]. Dedicated approaches to people with frailty using ambulance services have been introduced in some regions of the UK [12, 13] and elsewhere [14] but are not yet in widespread use. Initiatives to introduce routine frailty assessment of older patients have been the subject of preliminary or small-scale research studies [15, 16]. To date, there has been little prospective or consistent application of frailty assessment in UK paramedic practice, guidelines or training. However, recognising and measuring frailty in the ambulance setting could support decision making regarding escalation of care, on-scene management and conveyance to ED, and communication of patient status at the point of transfer between care settings.

This study aimed to measure frailty in people aged ≥ 50 in an ambulance setting (the phase of care delivered by a paramedic), using the Clinical Frailty Scale (CFS) [17], and describe the relationship between frailty, probability of conveyance to hospital, and duration spent by ambulance personnel on-scene.

## Methods

### Study design and setting

A cross-sectional observational study conducted between January and June 2021, reported in accordance with the Strengthening the Reporting of Observational Studies in Epidemiology (STROBE) guidelines [18]. The study was conducted at North East Ambulance Service (NEAS) NHS Foundation Trust, which is one of ten ambulance services in England. It covers North East England, serves a population of 2.71 million people across urban and rural areas and conveys patients to eight EDs [19].

### Selection of participants

Eligible patients were aged ≥ 50 years and attended by a study paramedic in response to an emergency call. Patients who required immediate treatment for a life-threatening condition (ambulance response priority category one calls) [20] were excluded. Patients with impaired consciousness (Glasgow Coma Scale (GCS) score < 15) [21] were also excluded.

Ambulance priority categories are assigned by means of a uniform approach in England and specify the urgency of a patient’s condition based on information gathered by call handlers during the initial emergency call [20]. The GCS assesses impaired consciousness using ocular, oral and movement-based tests. These tests were conducted by paramedics when attending to patients as part of routine data collection.

### Interventions

Study paramedics (*n* = 112) undertook an online training package in how to use the CFS. They then used routinely collected data and clinical judgement to assess the frailty status of eligible patients for the day of contact and for two weeks prior to the day of contact (to reflect the patient’s baseline health status and frailty) [22]. Baseline status is particularly relevant in clinical settings where health can change quickly [22].

### Measurements and outcomes

Duration at scene was measured using two distinct intervals. For patients who were not conveyed to hospital, this was the interval between the ambulance arriving at the patient’s location and the time that the ambulance crew become available for further jobs.

For patients conveyed to hospital, we used the interval between the time the ambulance arrived at the patient’s location and the time it departed for the hospital.

Anonymised patient demographic data, clinical characteristics (including National Early Warning Scores (NEWS2)) [23], conveyance to hospital status, and incident cycle times (time of arrival at scene, time of departure to hospital and time of availability for another call) were collected remotely from a report generated from the electronic patient care record (ePCR) system (ePCRs are completed by the paramedic for each incident and contain a standardised summary of the clinical episode). NEWS2 is a standardised, aggregate score derived from routinely collected physiological measurements, which grades the severity of acute illness in patients [23]. Scores of five or greater are associated with adverse clinical outcomes.

### Clinical Frailty scale (CFS)

The CFS is a clinical judgement-based frailty tool developed for the Canadian Study of Health and Aging [24]. It is used across the world as a tool to assess frailty and supported in the UK by the National Institute for Health and Social Care Excellence [25] and the British Geriatric Society [26]. The scale has been found to reliably identify frailty in individuals requiring emergency care [27, 28] and combines assessment of comorbidity, cognitive impairment and disability, to stratify older adults into one of nine ordinal categories [17]. We follow previous analyses and define frailty as CFS ≥ 5 [29]. Baseline frailty is obtained by asking the patient/family what the patient’s capabilities were two weeks ago.

### Statistical analysis

Ordered logistic regression models were used to investigate the relationships between frailty and clinical, demographic and socioeconomic characteristics. Models were checked for compliance with the proportional odds assumption. The relationship between duration on scene, frailty status and clinical, demographic and socioeconomic characteristics, was studied using quantile regression on the median. Missing data on NEWS2 (2%) and time at scene (2%) were handled using multiple imputation by chained equations (Appendix A1).

Inverse probability weighting was used to account for differences in the probability of patients being included in our survey data (Appendix A2). Area deprivation was measured using the English Index of Multiple Deprivation (IMD). This combines metrics of income, employment, education, health, crime, barriers to housing and services, and the living environment to produce a relative ranking of each small area (average population 1500) of England [30]. Deprivation quintiles were calculated to produce a relative measure of deprivation using the IMD. Statistical analysis was conducted with *Stata 17*  [31] and *R v3.6.*3 [32].

The relationship between CFS and study month was checked as a sensitivity analysis. This indicated whether frailty assessments varied as paramedics became more familiar with the CFS or due to seasonal effects.

Ambulance attendances and hospital conveyance rates reduced during the first two months of the COVID-19 pandemic (March-April 2020) [33]. While our data was collected approximately one-year after this, patient and paramedic behavour may have been similarly influenced when COVID-19 case rates were high. Investigating whether CFS varied with study month provided a check on possible influences of COVID-19, as case rates within the study region varied by a factor of 26 over the study period [34].

## Results

### Descriptive characteristics

We observed 3,056 callouts over a six-month period (Table 1). Observed patients had a mean age (unweighted) of 78.1 (95% CI 77.7–78.5) years and the proportion of females (unweighted) was 57.2%. Weighted frailty prevalence was high (58.7% frail), and higher in urban than rural areas (59.9% of patients in urban areas, 53.0% in rural areas). Weighted mean CFS scores were similar for men (4.85, 95%CI: 4.72–4.97) and women (4.90, 95% CI: 4.80–5.01). Callout numbers were approximately proportionate to the population size in each deprivation quintile of North East England (Appendix A3).

**Table 1:** Baseline characteristics of patients by frailty status

**Characteristic**	Unweighted	Weighted	
	**Total (n)**	**Not frail (CFS < 5) (%)**	**Frail (CFS ≥ 5) (%)**
*All*	3056	41.3	58.7
*Gender*			
Female	1748	40.6	59.4
Male	1308	42.2	57.8
*Age*			
50–54	120	74.8	25.2
55–59	144	75.8	24.2
60–64	209	59.0	41.0
65–69	236	52.5	47.5
70–74	326	44.6	55.4
75–79	422	34.3	65.7
80–84	557	25.5	74.5
85–89	549	19.9	80.1
≥ 90	493	11.7	88.3
*Deprivation*			
1 (Most dep.)	1068	40.9	59.1
2	792	40.3	59.7
3	467	42.6	57.4
4	390	44.1	55.9
5 (Least dep.)	339	40.4	59.6
*Rurality*			
Urban	2435	40.1	59.9
Rural	621	47.0	53.0
*NEWS2*			
< 5	2594	42.8	57.1
≥ 5	404	32.1	68.0

Weighted mean NEWS2 was higher for people with frailty (2.1, 95%CI: 2.0-2.2) than those non-frail (1.5, 95% CI: 1.3–1.7).

**Fig. 1:**
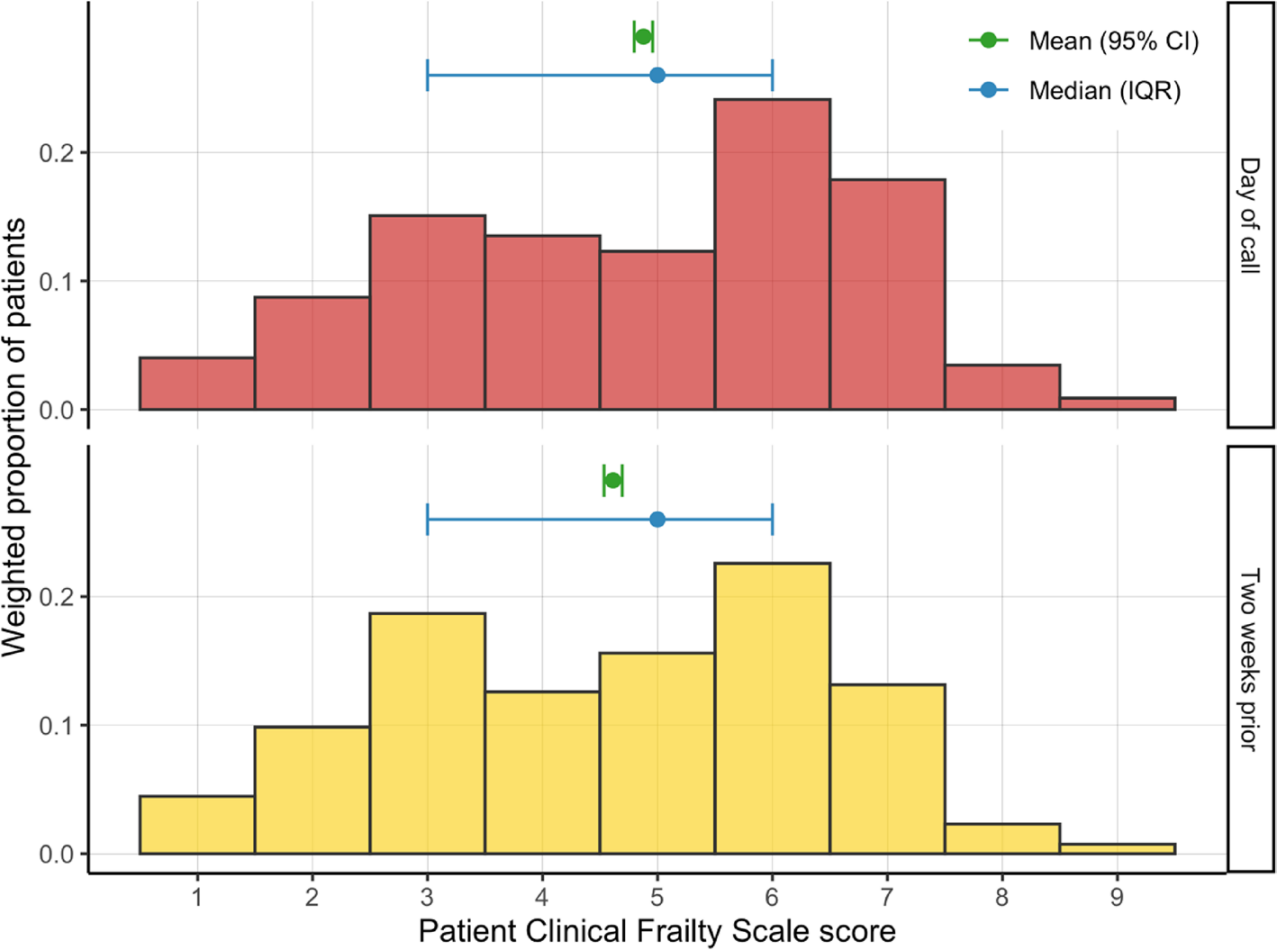
Weighted histogram of Clinical Frailty Scale scores assessed on the day of call and the estimated scores of the same patients for two weeks earlier

All patients had CFS information for the current attendance, but a proportion (1282, 42.0%) were missing a baseline frailty score (the estimated CFS score two weeks prior to contact). In patients with baseline frailty scores, the weighted *median* CFS score on the day of the ambulance attendance and the baseline scores were similar (median CFS = 5 [IQR:3–6] in both cases), although the weighted *mean* CFS score was higher on the day of attendance (mean CFS = 4.88 [95%CI:4.80–4.96]) than the baseline score (mean CFS = 4.61 [95%CI:4.53–4.69]) (Fig. 1). Of patients with baseline frailty scores (*n* = 1774, 58.0%), 23.6% (*n* = 419) were estimated to have had a change in CFS score over the two-week period, of which nearly all (*n* = 405, 96.7%) increased.

### Individual and area effects

The likelihood of a patient being assessed as frail increased with age, NEWS2 ≥ 5 and deprivation (Table 2). People in rural areas were less likely to be assessed as frail, compared to urban areas. Gender had no significant effect (at 95% confidence interval) on frailty among ambulance patients.

**Table 2:** Assessment of patients as frail (vs. not frail)

Characteristic	Odds ratio (95% confidence interval)		
*Individual characteristics*			
Age category (ref: 50–54)			
55–59	1.0 (0.5–1.9)		
60–64	2.3 (1.3–3.9)		
65–69	2.8 (1.6–4.7)		
70–74	4.2 (2.5–6.9)		
75–79	6.6 (4.0-10.8)		
80–84	10.4 (6.3–17.0)		
85–89	14.4 (8.7–23.7)		
90+	27.8 (16.3–47.3)		
Male	1.0 (0.8–1.2)		
NEWS2 ≥ 5	1.9 (1.4–2.6)		
*Area characteristics*			
Deprivation quintile (ref = 1, most deprived))			
2	0.9 (0.7–1.1)		
3	0.7 (0.6-1.0)		
4	0.6 (0.5–0.9)		
5 (least deprived)	0.7 (0.5–0.9)		
Rural	0.6 (0.5–0.8)		
Baseline	0.4 (0.2–0.6)		

We did not observe any statistically or clinically significant seasonal effects or variation in patient CFS scores over the study period (Appendix A4).

### Time with patient and conveyance

Most callouts (2275, 74.4%) resulted in conveyance to hospital, and weighted median duration at scene was 47.0 min (interquartile range 34.0–67.0 min).

Duration on scene for frail patients was on average nine minutes longer than for non-frail patients (8.2 min, 95%CI: 5.4–11.0), after adjusting for NEWS2, age, gender, area deprivation and rurality (Table 3). Higher NEWS2 was associated with decreased time at scene (-4.1 min, 95%CI: -7.4 – -0.9 if NEWS2 ≥ 5).

**Table 3:** Time at scene and odds ratio of conveyance to hospital. Times given relative to baseline time (39.1 min)

Characteristic	Times (mins)		Odds ratio	
*Individual characteristics*				
Frail	8.2 (5.4–11.0)		0.75 (0.60–0.94)	
Age (ref: 50–54)				
55–59	0.9 (-8.6–10.4)		0.94 (0.49–1.79)	
60–64	1.0 (-7.1–9.0)		0.77 (0.43–1.37)	
65–69	3.4 (-4.9–11.7)		1.16 (0.64–2.11)	
70–74	2.5 (-5.6–10.6)		0.71 (0.41–1.23)	
75–79	4.4 (-3.5–12.3)		0.82 (0.48–1.41)	
80–84	5.6 (-2.1–13.4)		0.76 (0.45–1.30)	
85–89	3.1 (-5.0–11.1)		0.87 (0.51–1.49)	
90+	5.9 (-2.6–14.3)		0.73 (0.42–1.25)	
Male	-3.3 (-5.9 – -0.7)		1.03 (0.85–1.25)	
NEWS2 ≥ 5	-4.1 (-7.4 – -0.9)		5.46 (3.26–9.14)	
*Area characteristics*				
Deprivation (ref: Most)				
2	4.2 (1.0–7.4)		1.08 (0.84–1.38)	
3	4.0 (0.1–8.0)		1.35 (1.01–1.80)	
4	0.4 (-4.0–4.8)		1.37 (1.01–1.86)	
5 (Least)	1.8 (-2.9–6.5)		1.18 (0.87–1.61)	
Rural	2.2 (-0.8–5.2)		0.91 (0.72–1.15)	
Baseline	39.1 (31.6–46.6)		3.36 (2.05–5.51)	

Frail patients were 25% less likely to be conveyed than non-frail patients (OR: 0.75, 95% CI: 0.60–0.94, Table 3).

Patients with NEWS2 ≥ 5 had an odds ratio (OR) of 5.5 (95% CI: 3.3–9.1) of conveyance. Other covariates (age, gender, area deprivation and rurality) were not associated with changes in the odds of conveyance.

When paramedics assessed a patient’s CFS to have increased over the past two weeks, their odds of conveyance also increased (OR: 2.2 [95% CI 1.5–3.2], relative to no increase in CFS) after adjusting age, gender, NEWS2, area deprivation and rurality (Appendix A5).

## Discussion

In this observational study of people aged ≥ 50 using an ambulance service, a large proportion of patients (58.7%) were frail (CFS ≥ 5). Frailty prevalence increased with advancing age and deprivation. Ambulance crews spent more time attending to people who were frail compared to people who were not. However, frail people were less likely to be conveyed to hospital.

Frailty is not well described in older populations using the ambulance service, and to our knowledge there are no published studies reporting frailty in this setting. Published estimates of community frailty prevalence vary, reflecting heterogeneity in clinical settings, population characteristics and frailty measures.

The English Longitudinal Study of Ageing (ELSA) provides data for several frailty metrics. Analyses of ELSA data have generated estimates of frailty prevalence ranging from 14% of adults aged ≥ 60^6^ to 8% among adults aged ≥ 50 [35]. The latter study also found higher prevalence of frailty in both the most deprived areas and urban areas of England. An analysis using primary care data, restricted to people aged ≥ 65, found 12% were moderately frail and 3% severely frail [36].

We did not observe the expected higher prevalence of frailty amongst women [37, 38] suggesting that older people who use the ambulance service are not representative of the older population in general.  

On average, paramedics spent eight minutes longer on-scene when attending frail patients compared to those who were not frail. Frailty is associated with medical complexity and instability [39], so a prolonged assessment may be justified. Furthermore, any additional community care for people not conveyed to hospital is likely to involve multiple providers and may be time consuming to arrange. In our study, frailty was associated with higher average NEWS2. However, frail patients were also more likely to be managed in the community or discharged on scene. Our data do not offer detailed insights into why patients with frailty were less likely to be conveyed to hospital. Possible influences on the paramedics’ clinical decision-making include advance care plans, availability of support from community services, or patients’ reluctance to go to hospital, particularly during the Covid-19 pandemic. Nevertheless, our study suggests a significant proportion of ambulance service time is spent attending frail patients who ultimately do not require conveyance to hospital.

### Limitations

This study used a pragmatic design to collect information on patient frailty in the ambulance setting, and is amongst the first to measure frailty and its association with key outcomes in this setting. Paramedic allocation to calls was random, but decisions to record frailty were not, as paramedics volunteered to enrol in the study.

To address the potential for selection bias, we reweighted our sample of callouts to match the population of eligible callouts on factors known to be associated with frailty. Future studies in this setting should utilise systematic random sampling strategies to ensure accurate estimates of frailty prevalence.

When a patient is acutely unwell, knowledge of usual levels of functional ability and daily activities helps clinical decision-making. Information from the patient, family or care records can be used to allocate a baseline CFS reflecting the patient’s status two weeks before the current clinical episode [40]. We were missing a proportion of these data (42% across all callouts), which suggests that generating this assessment is challenging in the ambulance setting.

The non-missing baseline scores may be subject to recall bias as patients (especially those requiring attendance by a paramedic) may not accurately recall health and status information over this time frame, and cognitive and hearing impairment are common in this population.

Further research is needed to understand the barriers to obtaining these data in clinical practice. Additional training for ambulance clinicians (to assess frailty over time) and enhancing access to primary care records (which are likely to hold data sufficient to generate historical or longitudinal frailty status) are possible solutions.

Study paramedics received training on how to use the CFS online, rather than face to face due to the coronavirus pandemic. There are limitations associated with online training; study paramedics had no opportunity to discuss any aspect of their learning, or clarify potential misunderstandings regarding the CFS. We were unable to judge the study paramedics knowledge of the CFS following completion of the training package, nor determine their competency to apply it in clinical practice. However, similar online frailty training packages have been rated as effective, feasible, and met with high satisfaction from users [41].

COVID-19 is likely to have influenced decisions on conveyance to hospital, particularly with older patients involved in the study. Ambulance conveyance rates to ED’s varied significantly early in the pandemic [33]. However, given the high conveyance rate observed in this study any change in patient or clinician behaviour due to the pandemic appears limited.

Study data were collected during a six-month period. We did not observe any meaningful or systematic variations in frailty scores over this time in our sensitivity analyses. However, it is important to acknowledge that some of the seasonal effects associated with variation in ambulance call patterns [42] may have influenced the characteristics of the study participants.

### Interpretation

In the future, paramedics are likely to have increasing involvement with a growing population of older people living with frailty. Enhancing clinical understanding and recognition of frailty improves patient outcomes and patient-centred care in acute settings [43].

Routine frailty assessment by the ambulance service could support clinical decision making and provision of appropriate community care for those not conveyed to hospital. This study suggests that paramedics would require more training to be confident in frailty assessment. Improved record sharing between different care settings could allow access to automatically generated frailty scores such as the hospital frailty index [44] or electronic frailty index [35] and support routine collection of frailty data in the ambulance setting. Longitudinal profiles of frailty at individual and population level could enable ambulance services to be more responsive to the demands of an ageing population.

## Conclusion

In summary, frailty prevalence is high in people aged ≥ 50 using the ambulance service. As the population ages, ambulance services will need to be trained and resourced to assess and manage frail patients in order to effectively meet their needs. However, access to appropriate community health and social care services is also essential to optimise patient experiences and reduce demand on ambulance and hospital services.

## Appendix

### A1 missing data

Missing data was generally low, with 2% missing for NEWS2 and time at scene. Multiple imputation by chained equations imputes values for missing data based on the distributions of observed data. Multiple sets of imputed data are generated, producing a credible distribution of values for missing data.

Here, missing data was imputed using multiple imputation by chained equations, using the complete data available for age, gender, area deprivation quintile, CFS score on day of attendance, rurality, if the patient was conveyed to hospital and whether the patient was located in a care home. Forty imputations were calculated [45]. Imputed values for time at scene were sampled by predictive mean matching using a pool of 10 donors [46]. Imputed values for NEWS2 ≥ 5 were sampled by logistic regression.

Missingness was greater for frailty two weeks prior to ambulance attendance (42%). This variable was not imputed due to high missingness. Analysis using this data was limited to patients for whom it was recorded.

### A2 weights

Table 4 shows the odds ratio of patients in the population data set being included in the study data set. Older and female patients were more likely to be included, while those with NEWS ≥ 5 had lower odds. Deprivation did not appear to have a significant effect on inclusion in the study.

**Table 4:** Odds ratios of being included in study sample, based on all eligible ambulance call outs during the study period

Characteristic	Odds ratio (95% Confidence Interval)	
Age (ref: 50–54)		
55–59	1.11 (0.86–1.45)	
60–64	1.56 (1.23–1.98)	
65–69	1.77 (1.40–2.24)	
70–74	1.95 (1.56–2.44)	
75–79	2.34 (1.88–2.90)	
80–84	2.70 (2.19–3.34)	
85–89	2.98 (2.41–3.67)	
≥ 90	3.26 (2.63–4.04)	
Female	1.08 (1.00-1.16)	
Deprivation quintile (ref: 1, Most)		
2	1.12 (1.02–1.24)	
3	1.13 (1.00-1.27)	
4	1.04 (0.91–1.17)	
5 (Least)	1.09 (0.96–1.24)	
NEWS2 ≥ 5	0.74 (0.64–0.86)	
Rural	1.11 (1.01–1.22)	
Care home resident	1.10 (0.99–1.23)	
Ambulance priority category (ref: 2)		
3	1.52 (1.40–1.65)	
4	0.71 (0.59–0.85)	
Baseline	0.01 (0.01–0.02)	

Survey weights were generated with inverse probability weighting. We used data from all eligible ambulance calls (patient aged ≥ 50, Glasgow Coma Scale score = 15 and not ‘category 1’ 999 calls) in the North East Ambulance Service region during the survey period (*n* = 86,311) to produce a population dataset to generate weights. The population dataset was sourced from electronic emergency call logs

Weights accounted for age, gender, Index of Multiple Deprivation quintile, rurality, NEWS2 of patients, whether the patient was located at a care home and the ambulance priority category of the call

A small number of patients in our study were not included in the population dataset due to the patient’s age not being recorded in the electronic emergency call logs (45), or the patient being initially classified as category 1 (175). This is because some patients whose urgency category was downgraded from category 1 once a study paramedic arrived on scene were included in our study. Patients in the study dataset, but not included in the population dataset, were retained in our analysis but assigned a neutral weighting of 1.

### A3 area deprivation (Table 5)

**Table 5:** Percentage of small areas (Lower Super Output Areas) in the North East of England in each deprivation quintile. Deprivation measured by English Index of Multiple Deprivation (2019)

Deprivation quintile	Percentage of small areas (LSOAs) in the North East of England in each deprivation quintile	Number (percentage) of calls to each deprivation quintile (unweighted).
1 (Most)	35	1068 (34.9)
2	22	792 (25.9)
3	14	467 (15.3)
4	15	390 (12.8)
5 (Least)	14	339 (11.1)

### A4 study month

**Fig. 2:**
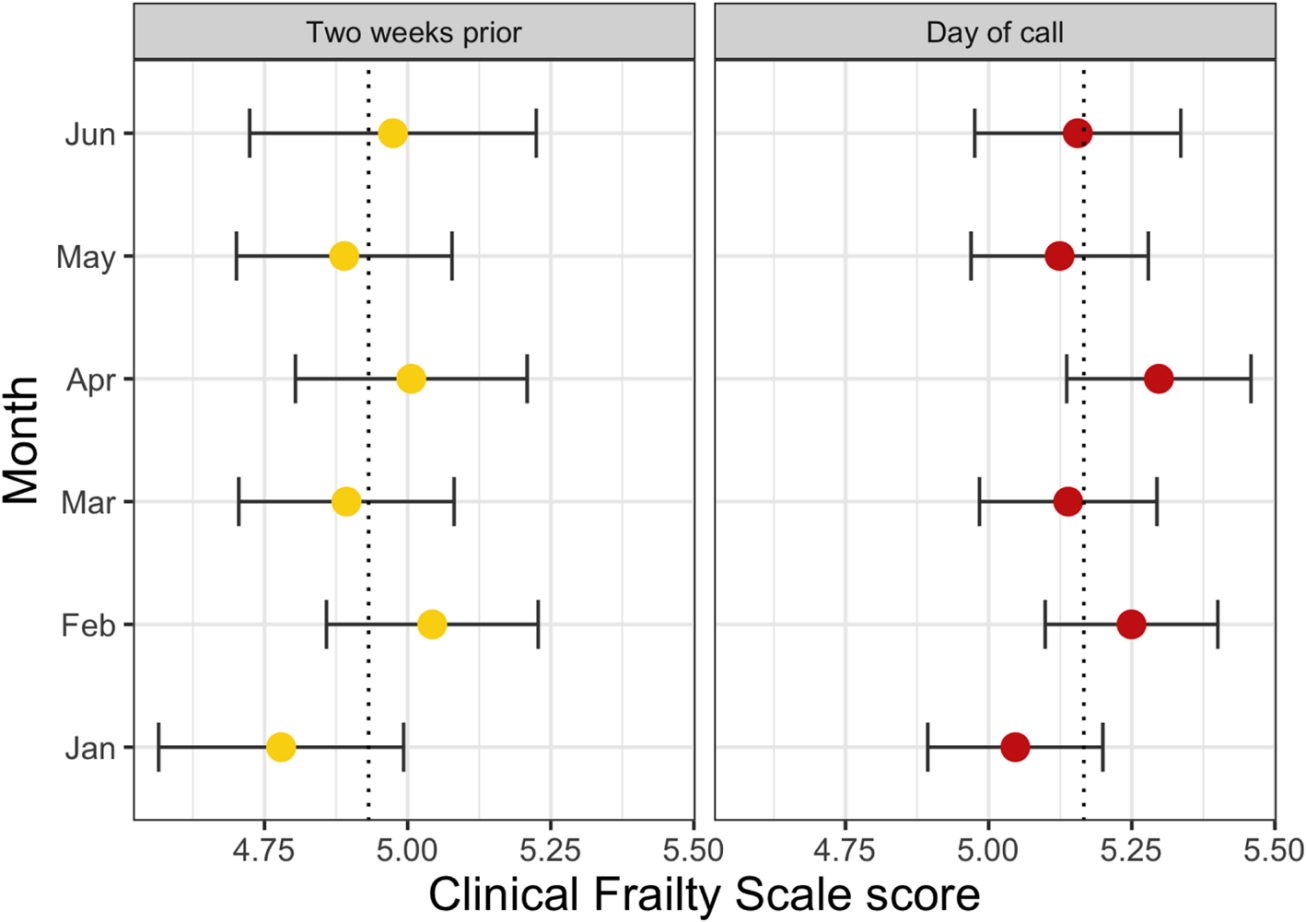
Patient Clinical Frailty Scale scores (mean and 95% confidence intervals) for each month of the study. CFS scores on the day of ambulance call and the estimate of patients’ score two weeks prior to the call are shown. Dashed line indicates the mean CFS score over the six-month period

CFS scores did not vary significantly over the course of the study (Fig. 2). Large changes in the NEAS region’s COVID-19 case rate over the study period did not appear to be associated with patient CFS scores; the seven-day rolling case rate per 100,000 people in the North East of England dropped from 494.4 (4 January 2021) to 19.3 (16 May 2021) before increasing to 495.6 (30 June 2021) over the study period [https://coronavirus.data.gov.uk].

### A5 conveyance with increased frailty score (Table 6)

**Table 6:** Odds ratios of a patient being conveyed to hospital if their Clinical Frailty Score increased in the two weeks prior to their ambulance call

Characteristic	Odds ratio	
Frailty score increased	2.16 (1.48–3.15)	
NEWS2 ≥ 5	5.24 (2.54–10.79)	
Deprivation (ref: 1, Most)		
2	1.09 (0.77–1.54)	
3	1.32 (0.89–1.96)	
4	1.19 (0.79–1.78)	
5 (Least)	1.38 (0.90–2.13)	
Age (ref: 50–54)		
55–59	0.88 (0.38–2.03)	
60–64	0.76 (0.37–1.56)	
65–69	0.94 (0.45–1.97)	
70–74	0.62 (0.31–1.22)	
75–79	0.59 (0.30–1.16)	
80–84	0.54 (0.28–1.03)	
85–89	0.66 (0.34–1.28)	
90+	0.56 (0.29–1.10)	
Male	1.14 (0.87–1.50)	
Rural	0.94 (0.69–1.30)	
Constant	2.82 (1.71–4.63)	

## Data Availability

The datasets used and/or analysed during the current study are available from the corresponding author on reasonable request.

## Abbreviations

NHS: National Health Service
CFS: Clinical Frailty Scale
STROBE: Strengthening the Reporting of Observational Studies in Epidemiology guidelines
NEAS: North East Ambulance Service NHS Foundation Trust
GCS: Glasgow Coma Scale
NEWS2: National Early Warning Score 2
ePCR: electronic Patient Care Record
IMD: Index of multiple deprivation
ELSA: English longitudinal study of ageing
